# Use of Posttranscription Gene Silencing in Squash to Induce Resistance against the Egyptian Isolate of the* Squash Leaf Curl Virus*

**DOI:** 10.1155/2016/6053147

**Published:** 2016-03-13

**Authors:** Omnia Taha, Inas Farouk, Abdelhadi Abdallah, Naglaa A. Abdallah

**Affiliations:** ^1^Agricultural Genetic Engineering Research Institute, ARC, Cairo 12613, Egypt; ^2^Department of Genetics, Faculty of Agriculture, Cairo University, Cairo 12619, Egypt

## Abstract

*Squash leaf curl virus* (SqLCV) is a bipartite begomovirus affecting squash plants. It is transmitted by whitefly* Bemisia tabaci* biotype B causing severe leaf curling, vein banding, and molting ending by stunting. In this study full-length genomic clone of SqLCV Egyptian isolated and posttranscriptional gene silencing (PTGS) has been induced to develop virus resistance. The Noubaria SqLCV has more than 95% homology with Jordon, Israel, Lebanon, Palestine, and Cairo isolates. Two genes fragment from SqLCV introduced in sense and antisense orientations using pFGC5049 vector to be expressed as hairpin RNA. The first fragment was 348 bp from replication associated protein gene (*Rep*). The second fragment was 879 bp representing the full sequence of the movement protein gene (*BC1*). Using real-time PCR, a silencing record of 97% has been recorded to* Rep/TrAP* construct; as a result it has prevented the appearance of viral symptoms in most tested plants up to two months after infection, while construct containing the* BC1* gene scored a reduction in the accumulation of viral genome expression as appearing in real-time PCR results 4.6-fold giving a silencing of 79%, which had a positive effect on symptoms development in most tested plants.

## 1. Introduction

The family Cucurbitaceae comprises many vegetable and fruit crops including cucumbers, squashes, luffas, melons, and watermelons. Squash (*Cucurbita pepo*) is a major vegetable crop grown all over the world from the cold region to the tropical one [1]. The world production of squash reported by FAO statistics for 2002 exceeded 17.7 million tons from 2.4 million ha. China is the most important producer on the world, while in Africa Egypt is one of the 10 top producers ranking as the eighth country from the world production of squash depending on last report of FAO 2012.

There are many pests that significantly affect the yield and fruit quality of squash. Among these diseases, viral diseases are more important as they cause heavy economic losses. There are more than 35 viruses that have been isolated from the family Cucurbitaceae [2].

Geminiviridae represents the largest family of plant DNA viruses that infect a broad range of plants causing severe losses in the yield of economically important crops within tropical and subtropical regions. Geminiviruses are single-stranded circular DNA viruses that cause severe damage to wide range of economically important crops worldwide [3]. They are characterized by twinned icosahedra particles and have emerged globally as one of the most economically important pathogens.* Begomovirus* is the largest genus of the Geminiviridae family whose genus has two types classified depending on their genome into monopartite and bipartite. Bipartite begomoviruses genome has either one or two circular single strand DNA named components A and B; each one of them has its specific role in the virus life cycle [4]. Component A contains 5 genes named replication associated protein (*Rep* or* AC1*), replication enhancer protein (*REn* or* AC3*), transcription activator protein (*TrAP* or* AC2*),* AC4*, and coat protein (*CP* or* AV1*). Component B contains 2 genes named movement protein (*MP* or* BC1*) and nuclear shuttle protein (*NSP* or* BV1*) [5].* NSP* and* MP* genes play significant role in virus movement either from the nucleus to the cytoplasm or from cell to cell through plasmodesmata, respectively.* Squash leaf curl virus* (SqLCV) is transmitted by its whitefly vector* Bemisia tabaci* and it induces severe stunting and leaf curling in squash plants and causes severe losses in cucurbit production. The first record of SqLCV in the Middle East was in Israel in 2003; subsequently it has spread to neighbor countries such as Egypt [6], Jordan [7], Palestine [8], and Lebanon [9]. The spreading of the SqLCV within Egypt was quickly as it took 5 years to spread from one governorate to another [6, 10, 11]. Restriction enzymes of RCA products combined with a cloning strategy showed being a very efficient method to obtain full genome sequences. This technique allowed the easy detection, cloning, and sequencing of a mixed infection and the identification of the respective begomoviruses on the same sample [12–14].

The small interference RNA (siRNA) or posttranscriptional gene silencing (PTGS) occurs naturally in plants and acts as an antiviral defense mechanism. The siRNA has been widely used as a method to engineer resistance against viruses in plants, including nuclear-replicating geminiviruses, which have no double-stranded RNA phase in their replication cycle. The produced virus specific siRNA from the breakdown of dsRNA induces the degradation of homologous mRNA sequences, resulting in hindering the virus form expressing its genes, thus limiting virus replication process [15].

In this study the two viral components of the Egyptian SqLCV Noubaria isolate were cloned and their sequences were submitted to the GenBank, from the cloned viral genome two regions, one from early and one from late expressed genes which were used to trigger PTGS against SqLCV using agroinfiltration as a transient experiment method. The first region is located at the overlapped region between* Rep* and* TrAP* genes. The second one represents the gene* BC1*, which has an important role in virus spreading from cell to cell causing systemic infection.

## 2. Materials and Methods

### 2.1. Cloning Viral Genome

Geminivirus-infected squash plants were collected from different cultivated open fields of Noubaria village at Beheira Governorate. The collected plant samples showed symptoms of* Squash leaf curl virus* (SqLCV) such as severe systemic stunting and leaf curling. Nonviruliferous whiteflies (*Bemisia tabaci*) were obtained from rearing whiteflies on healthy cotton plants in an isolated insectary for at least a month. Whiteflies were fed for a month on infected squash plants collected from the field to acquire the virus and used to infect healthy squash plants.

Total DNA from infected plants was extracted by using CTAB method [16]. Preliminary PCR for viral detection was carried out using the degenerate primers V324 (+) primer 5′-GCCYATRTAYAGRAAGCC MAG-3′ and C889 (−) primer 5′-GGRTTDGARGCATGHGTACATG-3′ specific for whitefly transmitted geminiviruses [17] to amplify 570 bp fragment. For cloning the complete viral genome, the amplified method was used. Total DNA extracted from infected squash plants was subjected to *φ*29 amplification essentially according to the manufacturer's instructions (Fermentas). The amplified products were denatured at 65°C for 10 min and run on a 1% agarose gel to confirm amplification. The ampliPhi product was digested independently with selected restriction enzymes, that is,* Eco*RI,* Hin*dIII,* Bam*HI,* Sal*I,* Sac*I,* Pst*I,* Xba*I,* Bgl*II,* Kpn*I,* Nco*I,* Xma*I,* Xho*1,* Sma*I,* Sph*I, and* Swa*I (Fermentas, fast digest) and separated in an agarose gel. The enzymes that could cut at a single site in the genome generating genome size fragment was selected for further cloning in pBluescript II SK (−). Sequencing was carried out and was subjected to GenBank (GenBank accession A: KP795975; B: KP795976).

### 2.2. Primer Design and PCR Amplification

To assess silencing effect of the selected viral fragments potentially useful in the control of SqLCV, two fragments were selected from the viral sequences to cover partial* Rep/TrAP* and full* BC1* genes. Two sets of primers were designed to amplify the selected regions by using Vector NTI® Software.* Rep/TrAP* primer sequences are 5′-CCCACATAATTACTTGAGCGGCCAT-3′ and 5′-TTAGCAATCCTGTGCTGTGCTTTGA-3′, while* BC1* gene primer pairs are 5′-ATGGGTTCTCAATTAGTTCCACCC-3′ and 5′-GCTTAGGGATTTTGGAGTGCTCGGG-3′. Restriction sites* Xho*I and* Xma*I added to the forward primers and* Asc*I and* Bam*HI to the reverse primers to facilitate cloning in sense and antisense forms in the binary vector pFGC5041 within the intron region. The expected sizes of the selected regions were 348 bp and 879 bp for cloned viral genome to amplify the selected region. The sizes of the selected fragments were 348 bp and 879 bp for* Rep/TrAP* and* BC1* genes, respectively. PCR was carried out using Flexi GoTaq® DNA polymerase (Promega, USA). Cloned viral genome was used as a template in PCR reaction with a concentration of 100 ng, 5 *μ*L of 5x Flexi GoTaq buffer, 2 *μ*L of 25 mM MgCl_2_, 0.5 *μ*L of 10 mM dNTPs, 0.5 *μ*L 10* p*mol of each primer, 0.25 *μ*L GoTaq enzyme, and ddH_2_O to reach a total volume of 25 *μ*L; the amplification was carried out in a BioRad T100 USA. The reaction was performed with an initial cycle at 94°C for 4 min, followed by 30 cycles of denaturation step at 94°C for 1 min, annealing at 50°C for 30 sec, and an extension cycle at 72°C for 1 min followed by a final cycle at 72°C for 7 min.

### 2.3. Cloning of Silencing Fragments

The amplified fragments were cloned initially into pGEM®-Teasy Vector System; subsequently ReP/TrAP and BC1 fragments were digested using* Xho*I and* Xma*I and* Asc*I and* Bam*HI for cloning in pFGC5041 silencing binary vector (supplied by Richared Jorgensen, University of Arizona, USA). Each fragment was cloned into the expression vector pFGC5041, in which the transgene is cloned in both orientations separated within the ChsA intron and under the control of the 35S promoter and the OSC terminator. The cloning of amplified fragments has been carried out in two steps by using the inner restriction sites on the primers. Double digestion with FastDigest enzymes* Xma*I and* Bam*HI was used for the sense orientation into the plant vector pFGC5041; the outer restriction enzymes pair* Xho*I and* Asc*I were used to clone the antisense orientation. All enzymes used for cloning were FastDigest restriction enzymes supplied from Thermo Scientific*™*.

After confirming the presence of the gene fragments by sequencing, pFGC-Rep/TrAP and pFGC-BC1 were transformed to electrocompetent* Agrobacterium* LB4404 by electroporation [18]. Electroporation was carried out using BioRad Gene Pulser set at 25 *μ*F, 200 Ω, and 2.5 kV. Transformed bacteria were detected by colony PCR, using specific primers for each construct.

### 2.4. Transient Expression of Silencing Fragments in Squash Plants

Squash plants (*Cucurbita pepo* cv. Eskandrani) were grown in the biocontainment greenhouse in the Agriculture Genetic Engineering Research Institute (AGERI) at 28°C with 16 hr light/8 hr dark. For each treatment twenty plants were used. Plants at the stage of two true leaves were infiltrated with the recombinant* Agrobacterium*. Infiltration media were prepared as follows: 5 mL overnight culture was used to inoculate 50 mL of LB medium containing 1 : 1 kanamycin and 0.5 : 1 streptomycin and incubated overnight at 28°C. Upon centrifugation of the 50 mL overnight culture, it has been resuspended in infiltration media containing 10 mM MgCl_2_, 10 mM, MES, and 20 *μ*M acetosyringone and then was incubated at room temperature for 2 hr till its OD_600_ reached 2.0.* Agrobacterium* harboring sense/antisense Rep/TrAp construct and sense/antisense BC1 construct infiltrated employing needleless 5 mL syringe using the Syringe Spotted Technique developed by Johansen and Carrington [19]. After 1 week from infiltration viruliferous whiteflies feed on infiltrated plant for 48 hr inoculation access period. The treated plants were sprayed with imidacloprid (Confidor, Bayer, Leverkusen, Germany) to get rid of the whiteflies. Plants infiltrated with silencing constructs were assayed for positive inoculation with specific PCR primers for each fragment. PCR-positive plants were evaluated for their resistance to the viral infection.

### 2.5. Resistance Evaluation

Observation of symptom development was done in 30 days after inoculation. The accumulation of viral DNA in infected agrobacterium plants was determined by RT-qPCR using the (leaves weight: 0.5–1.01 g) total DNA extracted from upper leaves [20].

Primers for RT-qPCR were designed by using Integrated DNA Technologies website tools [21]. The fragment of* BC1* amplified by 5′-CGGATTTGACCAGTCTTCTCTC-3′ and 5′-GGGCACCGACAGTAAAGATAC-3′ as forward and reverse primer, respectively, resulted in fragment with length 102 bp. In case of* Rep/TrAP* a fragment with length 105 bp was produced by those primers 5′-TATCCAAGGTGCGATGTTCC-3′ as forward and 5′-ATGTTCCTCTCTGGCCATTC-3′ as reverse one. Annealing temperature was 60°C for both fragments, while 18s rRNA was used as internal control for gene expression. Squash plants were kept for 10 days after virus infection; then samples were collected for RT-qPCR detection, and total RNA was extracted using SV Total RNA Isolation System. cDNA was synthesized by using 3′ SMARTer*™* RACE cDNA Amplification Kit. The RT-qPCR was performed in a AB7300 Real-Time PCR System® (Applied Biosystems, USA) using SYBR®* Premix ExTaqII™* from TAKARA (Catalog number RR82SW, TAKARA, Japan); RT-qPCR analysis was performed as described by manufacturer. In real-time PCR, primers were tested using conventional PCR resulting in the amplification of fragments monitored at the expected size, where Rep primers gave a 105 bp fragment while BC1 gave a 102 bp fragment, with no primer dimer development.

## 3. Results

### 3.1. *Squash Leaf Curl Virus* Isolation

**Figure 1:**
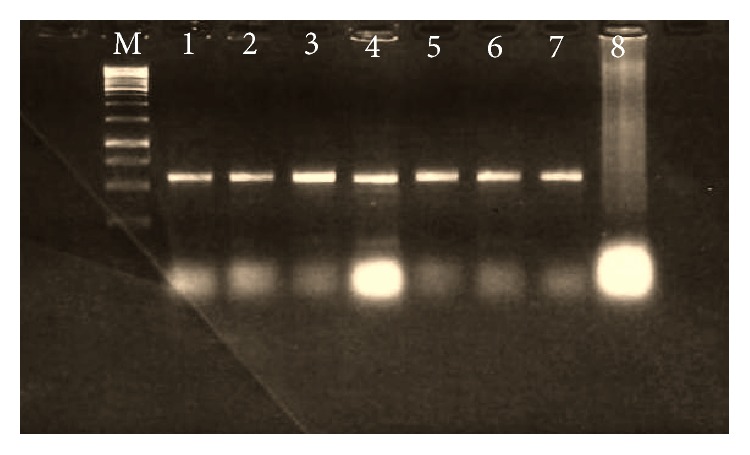
PCR amplification on squash plant samples with degenerate primers from* Cp* gene of geminivirus; M: Molecular Marker 1 kb Fermentas, lanes 1–6: squash plant samples, lane 7: positive control, and lane 8: negative control.

**Figure 2:**
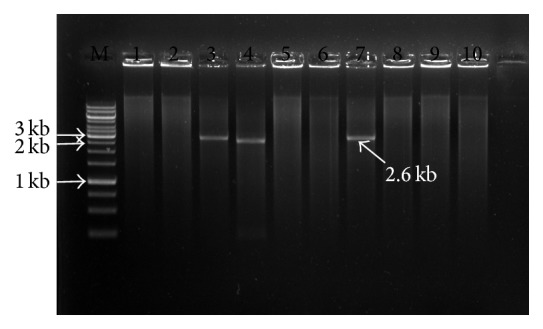
Restriction enzyme digestion of TempliPhi product of* squash leaf curl virus*; M: Marker 1 kb Fermentas, lane 1:* Sac*I, lane 2:* Xho*I, lane 3:* Eco*R I, lane 4:* Bam*H I, lane 5:* Not*I, lane 6:* Sma*I, lane 7:* Xma*I, lane 8:* Nco*I, lane 9:* Swa*I, and lane 10: undigested TempliPhi product.

**Figure 3:**
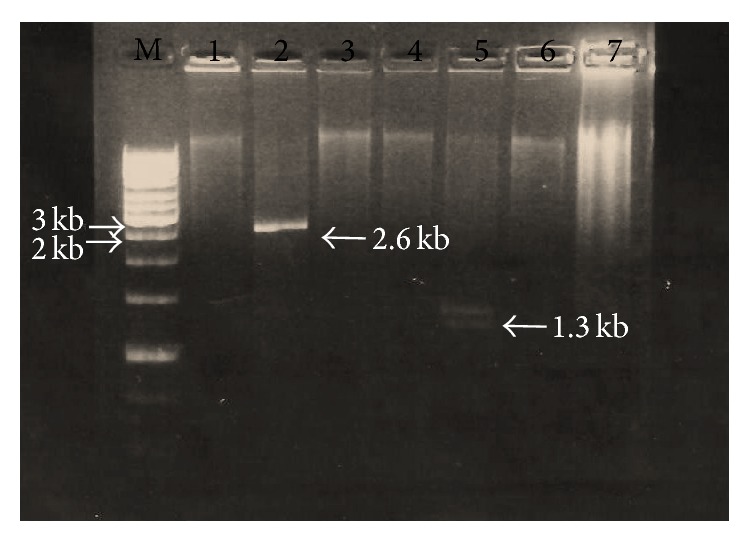
Restriction enzyme digestion of TempliPhi product of* squash leaf curl virus*; M: Marker 1 kb Fermentas, lane 1:* Not*I, lane 2:* Bgl*II, lane 3:* Pst*I, lane 4:* Hind*III, lane 5:* Xba*I, lane 6:* Kpn*I, and lane 7: undigested TempliPhi product.

All plant samples from different fields gave the expected band with PCR using the degenerate primer of* CP* core region, indicating that these samples were infected with geminivirus (Figure 1). The digestion of amplified products resulted in 4 unique cuts with enzymes* Eco*RI,* Bgl*II,* Bam*HI, and* Sal*I (Figure 2) while only* Xba*1 enzyme cut twice (Figure 3). Linearized fragments with the expected size were cloned in the corresponding site into the* E. coli* vector pSK^−^. Three constructed plasmids from cloning of the linearized genome with* Eco*RI,* Bam*HI, and* Sal*I were sent for sequencing and the sequences were confirmed by similarity search using BLAST at National Center for Biotechnology Information (NCBI). Results showed that* Eco*RI gave component B while* Sal*I and* Bam*HI gave component A.

Identity ratio showed high similarity results reaching 99% homology to component A with other SqLCV isolates. Component B showed less similarity than component A but the identity ratio is still high giving 99% homology with Cairo isolate (DQ285020.1), 98% homology with Palestine isolate (KC441466.1), Jordon isolates (JX444574.1 and JX131282.1), Israel isolate (HQ184437.1), and Lebanon (HM368374.1), 96% homology with Kaliobeya isolate (KJ579954.1), and 95% homology with California isolate (DQ285017.1) and Arizona isolate (DQ285018.1).

### 3.2. Generation of the RNAi Construct and Squash Plant Transformation

**Figure 4:**
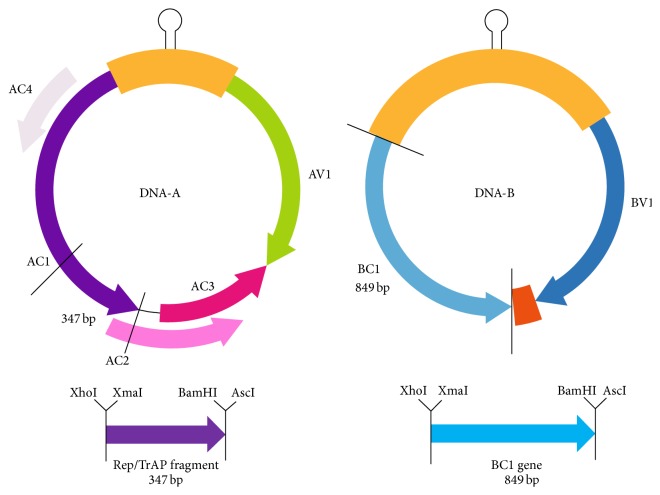
Illustration of silencing fragments* Rep/TrAP* and* BC1* sites on full genome of* squash leaf curl virus*.

**Figure 5:**
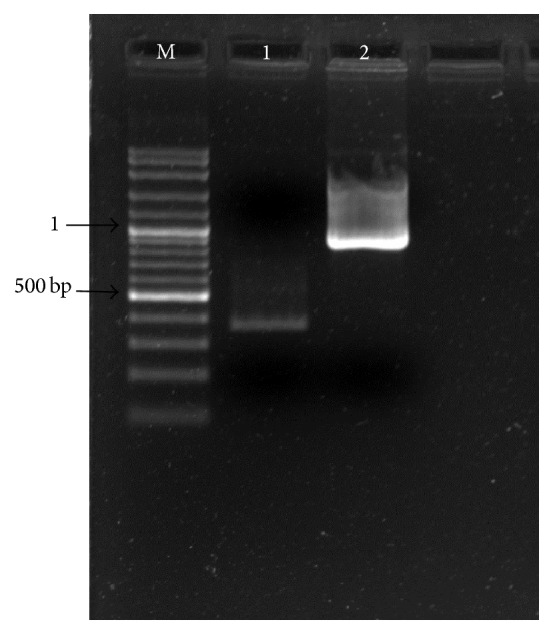
Molecular isolation for* squash leaf curl virus* genes using PCR with primers specific to* Rep* fragment and* BC1* gene; M: Marker 100 bp plus Fermentas, lane 1: Rep 3′ end fragment from base 701 to 1049 bp, and lane 2: full* BC1* gene.

**Figure 6:**
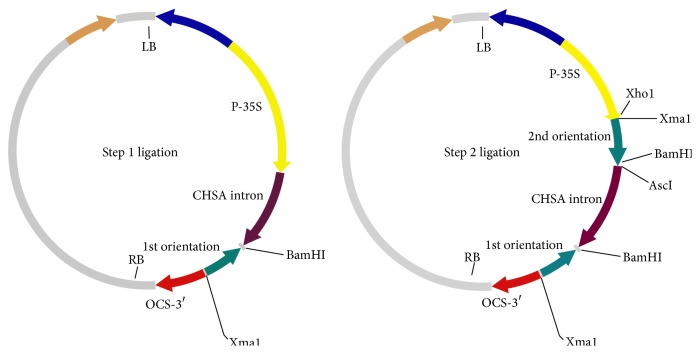
Schematic representation of the cloning strategy of the fragments* Rep/TrAP* and* BC1* in sense and antisense orientation into the binary vector pFGC5941 done in two steps using the inner and outer restriction enzyme sites.

To study SqLCV resistance using siRNA strategy on squash plants, two putatively effective fragments from the whole virus genome of the SqLCVA Noubaria were chosen to form short dsRNA structure for enhancing posttranscriptional gene silencing (Figure 4). The first fragment was designed targeting the* Rep/TrAP* with a length of 347 bp, while the second fragment was designed to contain the full* BC1* gene with length of 849 bp. PCR analysis was carried out to amplify both fragments giving the expected band size as showed in Figure 5. The insertion of the first fragment in the antisense orientation into the pFGC5041 vector was confirmed by restriction digestion using* Xho*I and* Asc*I enzymes, while* Bam*HI and* Xma*I enzymes were used to confirm the insertion of the second fragment in the sense orientation. The full* RNAi* cassette expression was driven by the e35S promoter and the* OSC 3*′ terminator (Figure 6). Positive clones of transformed* Agrobacterium* LB4404 with constructs pFGC5041-Rep and pFGC5041-BC1 were screened using colony PCR technique (data not shown).

To assess the expression of silencing fragments,* C. pepo* plant leaves were infiltrated in a transient manner. Plants were then left for a week and subsequently subjected to infection with viruliferous whiteflies raised on infected squash plants with SqLCV. To detect the presence of viral genome in infiltrated plants, PCR was carried out after inoculation on a daily basis. Primers of SqLCV coat protein gene were used in confirming PCR as a positive control. After the 7th day after infection noninfiltrated control plants gave positive results in PCR; however pFGC-Rep/TrAP and pFGC-BC1 infiltrated plants gave negative results in PCR. To monitor quantitative viral accumulation in infiltrated squash plants real-time PCR analyses were performed on infiltrated plants.

### 3.3. Evaluation of the Resistance Imparted by the* RNAi* Constructs by Transient Assay

#### 3.3.1. Monitoring Viral Symptoms

**Figure 7:**
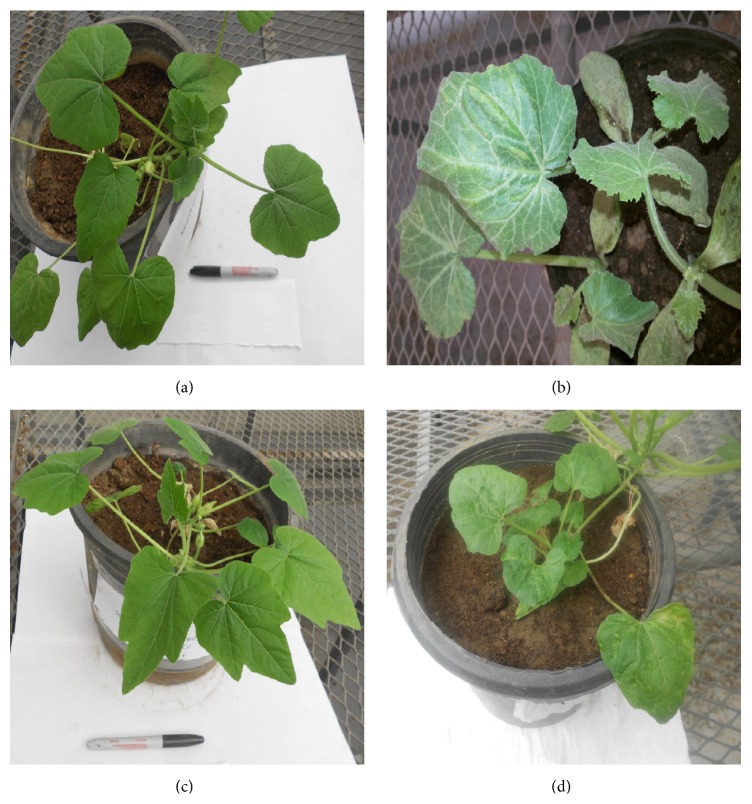
Symptoms evaluation after 2 months after infection. (a) Health squash plant, (b) infected plants with typical SqLCV symptoms, (c) infiltrated plant with pFGC-Rep/TrAP produced plants with mild symptoms, and (d) infiltrated plant with pFGC-BC1 produced plants with mild and moderate symptoms.

All noninfiltrated squash plants inoculated with viruliferous whiteflies carrying the SqLCV developed typical viral symptoms with severe yellowing, leaf curling, and a reduced leaflet size. Such plants ceased to grow, failing to flower and produce fruits, while the untreated plants remained the negative control (Figure 7(a)).

One month after inoculation by the pFGC-Rep/TrAP vector all plants were symptomless, while after two months only 1 plant of the total 5 plants gave mild symptoms = + (Figure 7(c)). Meanwhile, in the case of infiltrating the plants with pFGC-BC1 construct, one month after inoculation, plants produced 2 symptomless plants and 3 plants with mild symptoms. By the end of the second month only 1 plant showed being symptomless, 3 with mild symptoms, and 1 with moderate symptoms = ++ (Figure 7(d)) (Table 1). Control plants treated with empty pFGC5041 showed early symptoms after two weeks and severe symptoms after one month of inoculation (Figure 7(b)).

#### 3.3.2. Quantitative Screening of Viral Gene Expression

Quantitative analysis using RT-qPCR assay was carried out to study the level of the expression of the two viral genes,* Rep/TrAP* and* BC1* in the infiltrated squash. Total RNA extracted from infiltrated plants was used to synthesize cDNA and was subjected to RT-qPCR. In addition, RT-qPCR was performed from infected* C. pepo* plants as a positive control reaction and nontemplate DNA reaction that represent negative control which had* Ct* values equivalent to the total number of cycles used in the RT-qPCR experiment, resulting in curves that did not achieve the threshold level, thus indicating that these reactions contained no detectable viral DNA.

**Figure 8:**
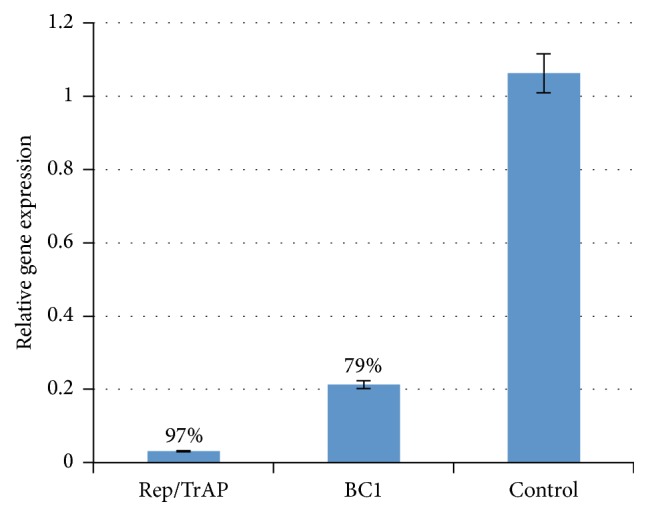
*siRNA*-mediated silencing of Rep/TrAP and BC1 was assessed using ΔΔCt method to determine relative gene expression from qPCR data using 18s rRNA as an endogenous reference gene. The cells exhibited* siRNA* of* Rep/TrAP* reduced the mRNA level by 97%, while* siRNA* of* BC1* reduced mRNA level by 79% compared with mRNA of control samples which fully expressed the* squash leaf curl virus* genes.

The level of virus detection was significantly lower in all infiltrated plants than in the control plants. Infiltrated plants with pFGC-Rep/TrAP construct showed significant shifting in* Ct* value compared with the control. Average* Ct* resulting from Re pFGC-Rep/TrAP treatment was 29.51 while an average* Ct* value of control plants was 24.29. In pFGC-Rep/TrAP samples the fold of change is calculated using the ΔΔCt and it showed a 32-fold decrease thus silencing percent was calculated to reach 97%. Also, pFGC-BC1 construct displayed a slight shifting in* Ct* values compared with that in the control. Treated samples* Ct* values average was 29.58 while control* Ct* values average was 27.48 resulting in a decrease 4.7-fold (Table 2). The calculated expression of* BC1* gene has decreased by 79% giving showing a good degree of plant resistance against the respected virus but not as efficient as pFGC-Rep/TrAP construct (Figure 8).

## 4. Discussion

SqLCV has become a major concern for cultivation of squash in the Middle East region. Thus, it is important to study the vast diversity of SqLCV and also the epidemic implications.

Small circular DNA is preferentially amplified by RCA, which makes the ssDNA genome of the geminiviruses ideal substrate for RCA. In addition, during viral replication, through complementary strand synthesis (CSR) of the circular ssDNA, rolling circle replication (RCR), and recombination-dependent replication (RDR), various DNA intermediates are produced [22]. In this work, the rolling circle amplification (RCA) technology was used for molecular cloning of the SqLCV genome, because of its simplicity, high sensitivity, and proofreading against misincorporation of nucleotides. It can be used to amplify sequences without the necessity of previous knowledge of it [12, 23]. The sequence of the SqLCV-Noubaria isolate obtained in this work showed high similarities to other isolates in the region. The low sequence variation observed among SqLCV isolates in the Middle East region was explained by Lapidot et al. [24]. They predict that uncontrolled movement of viruliferous whiteflies among countries causes viruses readily moved across country boundaries in this region, preventing strong genetic differentiation of these viruses among neighbor countries.

Studies on crops harboring virus resistance indicated the possibility of engineering the resistance by different transgene-based approaches [25]. During the past decade there has been considerable evidence for the successful using of siRNA to protect plants against viruses in plum, papaya, watermelon, and potato, which has led to the development of virus-resistant transgenic crops [26–30]. In this study the same technology has been used by the incorporation of sense and antisense fragments separated in pFGC5041 vector within the* CHSA* intron to be transcribed and hybridized with itself to form a hairpin structure [31]. However, RNA silencing degree induced in infiltrated plants could be diverse according to the gene used.

Two regions of* squash leaf curl* viral genome were selected for cloning, the first was* Rep/TrAP*, and the second was the full length of* BC1* gene. The selected region* Rep/TrAP* fragment was chosen according to its location representing the end of the* Rep* gene and the beginning of* TrAP* in overlapping manner, which may be putatively stronger than one of them alone as it could lead to the silencing of both of them, while in case of* BC1* gene it was selected because of its important function in systemic spreading of viral genome through the plasmodesmata, as its silencing attempt is expected to limit the infection and inhibit virus transmission from cell to cell.

The two constructed plasmids, pFGC-Rep/TrAP and pFGC-BC1, were prepared and used to infiltrate squash plants. Resistance was measured either by monitoring symptoms development or by real-time PCR. In real-time PCR, three replicates from each construct were tested, whereas detection of gene expression was measured by threshold cycle (*Ct*). Thus, higher* Ct* value means less amount of template in the sample, which indicates fewer titrations of virus particles. Depending on previous studies using the same virus,* 18s rRNA* was chosen to be used as the reference gene in real-time PCR [32]. To calculate relative gene expression ΔΔCt method was used, where target gene expression was normalized to reference gene expression within the same sample to correct any variation that could affect the study [33].

Results of Rep/TrAP fragment in real-time PCR have proved that there is a vital role for this fragment on the* Rep* gene expression as it has been decreased 32-fold and a silencing of 97% has been achieved through this construct. This significant result could be due to the key role of the* Rep* protein, which is the most essential protein for viral replication [34]. In addition, transcription activator protein gene is overlapped with* Rep* gene by 50 bp at its 3′ end; this overlapping has played a significant role in silencing of both genes and gave more efficient silencing percent; this could be clear because the transcription activator protein alone is acting as a gene silencing suppressor [35] so its early silencing may help the plants to resist higher.

The construct that contains the movement protein gene showed a degree of resistance accomplishment but being less efficient than the Rep/TrAP construct. A decrease in the virus quantity 4.7-fold has been achieved; this may be due to the late expression of this gene in the virus machinery or it may be due to the effect of its longer fragment size. In former studies fragments size showed efficient silencing ranged from ~110 to 550 bp [36, 37], while in the case of* BC1* gene used in this study it was 879 bp so it might be a factor that affect the silencing percentage.

Symptoms developed in all treated plants in each treatment completed the story with the real-time PCR results by giving ratio in plant resistance in case of pFGC-Rep/TrAP construct more than pFGC-BC1 construct.

We have demonstrated that hairpin constructs expressing either 348 bp of the* Rep/TrAP* gene or 879 bp for the* BC1* genes can control SqLCV. However, siRNA produced from using the* Rep/TrAP* construct proved to be more efficient in controlling the virus than* BC1* construct. It is clear from several studies that the* siRNA* strategy is more efficient at the 5′ and 3′ ends of the gene [36].

## 5. Conclusion

Both the RT-PCR and the symptoms screening at the greenhouse showed that the expression level of the* Rep* gene, as one of the early genes, could play the most significant role in silencing when it is employed as a posttranscriptional gene silencing and tool for controlling the* squash leaf curl virus.* The* BC1* gene could also give a degree silencing but we proved that it is less effective than the* Rep* gene. Therefore, the 3′ end fragment of the* Rep* gene for producing stable transformation should be used to measure effect of construct on the transgenic* C. pepo* plants and reach high level of resistance to SqLCV on large populations in the field.

## Figures and Tables

**Table 1:** Symptom severity of SqLCV during 2 months on infiltrated squash plants after SqLCV-viruliferous whiteflies feed on it.

Treatment	Total number of plants treated	Symptoms after 1 month				Symptoms after 1 month			
		No symptoms	+	++	+++	No symptoms	+	++	+++
pFGC-BC1 construct	5	2/5	3/5	0/5	0/5	1/5	3/5	1/5	0/5
pFGC-Rep/TrAP construct	5	5/5	0/5	0/5	0/5	4/5	1/5	0/5	0/5
pFGC5041 empty vector	5	0/5	2/5	3/5	0/5	0/5	0/5	1/5	4/5
−ve control untreated	5	5/5	0/5	0/5	0/5	5/5	0/5	0/5	0/5

**Table 2:** Quantitative real-time PCR estimation of viral expression levels for SqLCV in plants infiltrated by pFGC-Rep/TrAP and pFGC-BC1.

	*Rep*/*TrAP* gene				*BC1* gene			
	Ct of control	Ct of control reference	Ct of treatment	Ct of treatment reference	Ct of control	Ct of control reference	Ct of treatment	Ct of treatment reference
Replica 1	24.26	16.47	29.28	16.28	27.07	16.92	29.41	16.64
Replica 2	24.55	16.00	29.39	16.19	27.94	16.77	29.89	16.7
Replica 3	24.07	16.38	29.87	15.98	27.45	16.89	29.44	16.82
Average	24.29	16.28	29.51	16.15	27.48	16.86	29.58	16.72
ΔCt = Cts − Ctr^*∗*^	8.01		13.01		10.63		12.86	
ΔΔCt = Ctc − Ctt^*∗*^	5				2.23			
Fold of change = 2^ΔΔCt^	0.03125				0.213			
Silencing % = (1 − ΔΔCt) × 100	97%				79%			

^*∗*^Cts = Ct values of sample; Ctr = Ct value of housekeeping gene for the same sample; Ctc = Ct values of control; Ctt = Ct values of treatment.

## Abbreviations

SqLCV: * Squash leaf curl virus*
DNA: Deoxyribonucleic acid
RNA: Ribonucleic acid
Rep: Replication associated protein
*BC1*: Movement protein
*TrAP*: Transcription activator protein
*siRNA*: Small interference RNA
*PTGS*: Posttranscriptional gene silencing.

## References

[1] Moffat A. S. (1999). Plant pathology: geminiviruses emerge as serious crop threat. *Science*.

[2] Ozaslan M., Aytekin T., Bas B., Halil Kilic I., Didem Afacan I., Dag D. S. (2006). Virus diseases of cucurbits in Gaziantep-Turkey. *Plant Pathology Journal*.

[3] Mansoor S., Briddon R. W., Zafar Y., Stanley J. (2003). Geminivirus disease complexes: an emerging threat. *Trends in Plant Science*.

[4] Gutierrez C. (2002). Strategies for geminivirus DNA replication and cell cycle interference. *Physiological and Molecular Plant Pathology*.

[5] Sunter G., Bisaro D. M. (1992). Transactivation of geminivirus AR1 and BR1 gene expression by the viral AL2 gene product occurs at the level of transcription. *Plant Cell*.

[6] Farag A. G., Amer M. A., Amin H. A., Mayzad H. M. (2005). Detection of bipartite gemininviruses causing squash leaf curl disease in Egypt using polymerase chain reaction and nucleotide sequence. *Egyptian Journal of Virology*.

[7] Al-Musa A., Anfoka G., Misbeh S., Abhary M., Ahmad F. H. (2008). Detection and molecular characterization of Squash leaf curl virus (SLCV) in Jordan. *Journal of Phytopathology*.

[8] Ali-Shtayeh M. S., Jamous R. M., Husein E. Y., Alkhader M. Y. (2010). First report of *Squash leaf curl virus* in squash (*Cucurbita pepo*), melon (*Cucumis melo*), and Cucumber (*Cucumis sativa*) in the northern best bank of the Palestinian authority. *Plant Disease*.

[9] Sobh H., Samsatly J., Jawhari M., Najjar C., Haidar A., Abou-Jawdah Y. (2012). First report of Squash leaf curl virus in Cucurbits in Lebanon. *Plant Disease*.

[10] Idris A. M., Abdel-Salam A., Brown J. K. (2006). Introduction of the new world Squash leaf curl virus to Squash (*Cucurbita pepo*) in Egypt: a potential threat to important food crops. *Plant Disease*.

[11] El-Dougdoug K. A., Abd El-Kader H. S., Hamad I. A., Ahmed E. A., Abd El-Monem A. F. (2009). Identification of squash leaf curl virus (Egyptian Isolate). *Australian Journal of Basic and Applied Sciences*.

[12] Inoue-Nagata A. K., Albuquerque L. C., Rocha W. B., Nagata T. (2004). A simple method for cloning the complete begomovirus genome using the bacteriophage *φ*29 DNA polymerase. *Journal of Virological Methods*.

[13] Paprotka T., Boiteux L. S., Fonseca M. E. N. (2010). Genomic diversity of sweet potato geminiviruses in a Brazilian germplasm bank. *Virus Research*.

[14] Paprotka T., Metzler V., Jeske H. (2010). The complete nucleotide sequence of a new bipartite begomovirus from Brazil infecting Abutilon. *Archives of Virology*.

[15] Wesley S. V., Helliwell C. A., Smith N. A. (2001). Construct design for efficient, effective and high-throughput gene silencing in plants. *Plant Journal*.

[16] Doyle J. J., Doyle J. L. (1987). A rapid DNA isolation procedure for small quantities of fresh leaf tissue. *Phytochemical Bulletin*.

[17] Brown J. K., Idris A. M., Torres-Jerez I., Banks G. K., Wyatt S. D. (2001). The core region of the coat protein gene is highly useful for establishing the provisional identification and classification of begomoviruses. *Archives of Virology*.

[18] Sambrook J., Maniatis T., Fritsch E. F., Cold Spring Harbor Laboratory (1987). *Molecular Cloning: A Laboratory Manual*.

[19] Johansen L. K., Carrington J. C. (2001). Silencing on the spot. Induction and suppression of RNA silencing in the *Agrobacterium*-mediated transient expression system. *Plant Physiology*.

[20] Fulton T. M., Chunwongse J., Tanksley S. D. (1995). Microprep protocol for extraction of DNA from tomato and other herbaceous plants. *Plant Molecular Biology Reporter*.

[21] Eu.idtdna.com Integrated DNA Technologies—Home. https://eu.idtdna.com/site.

[22] Jeske H., Lütgemeier M., Preiss W. (2001). DNA forms indicate rolling circle and recombination-dependent replication of Abutilon mosaic virus. *The EMBO Journal*.

[23] Haible D., Kober S., Jeske H. (2006). Rolling circle amplification revolutionizes diagnosis and genomics of geminiviruses. *Journal of Virological Methods*.

[24] Lapidot M., Gelbart D., Gal-On A. (2014). Frequent migration of introduced cucurbit-infecting begomoviruses among Middle Eastern countries. *Virology Journal*.

[25] Collinge D. B., Jørgensen H. J. L., Lund O. S., Lyngkjær M. F. (2010). Engineering pathogen resistance in crop plants: current trends and future prospects. *Annual Review of Phytopathology*.

[26] Callaway A., Giesman-Cookmeyer D., Gillock E. T., Sit T. L., Lommel S. A. (2001). The multifunctional capsid proteins of plant RNA viruses. *Annual Review of Phytopathology*.

[27] Jan F.-J., Pang S.-Z., Fagoaga C., Gonsalves D. (1999). Turnip mosaic potyvirus resistance in Nicotiana benthamiana derived by post-transcriptional gene silencing. *Transgenic Research*.

[28] Ling K.-S., Zhu H.-Y., Gonsalves D. (2008). Resistance to *Grapevine* leafroll associated virus-2 is conferred by post-transcriptional gene silencing in transgenic *Nicotiana benthamiana*. *Transgenic Research*.

[29] Scorza R., Callahan A., Levy L., Damsteegt V., Webb K., Ravelonandro M. (2001). Post-transcriptional gene silencing in plum pox virus resistant transgenic European plum containing the plum pox potyvirus coat protein gene. *Transgenic Research*.

[30] Urcuqui-Inchima S., Haenni A.-L., Bernardi F. (2001). Potyvirus proteins: a wealth of functions. *Virus Research*.

[31] Agrawal N., Dasaradhi P. V. N., Mohmmed A., Malhotra P., Bhatnagar R. K., Mukherjee S. K. (2003). RNA interference: biology, mechanism, and applications. *Microbiology and Molecular Biology Reviews*.

[32] Abrahamian P. E., Abou-Jawdah Y. (2013). Detection and quantitation of the new world Squash leaf curl virus by TaqMan real-time PCR. *Journal of Virological Methods*.

[33] Haimes J., Kelley M. (2010). *Demonstration of a ΔΔ C q Calculation Method to Compute Relative Gene Expression from qPCR Data*.

[34] Elmer J. S., Brand L., Sunter G., Gardiner W. E., Bisaro D. M., Rogers S. G. (1988). Genetic analysis of the tomato golden mosaic virus II. The product of the AL1 coding sequence is required for replication. *Nucleic Acids Research*.

[35] Vanitharani R., Chellappan P., Pita J. S., Fauquet C. M. (2004). Differential roles of AC2 and AC4 of cassava geminiviruses in mediating synergism and suppression of posttranscriptional gene silencing. *Journal of Virology*.

[36] Rezk A. A., Abdallah N. A., Salam A. M. A., Nakhla M. K., Mazyad H. M., Maxwell D. P. (2005). Transgene-mediated RNA silencing of TYLCV genes affecting the accumulation of viral DNA in plants. *Arab Journal of Biotechnology*.

[37] Lin C.-Y., Tsai W.-S., Ku H.-M., Jan F.-J. (2012). Evaluation of DNA fragments covering the entire genome of a monopartite begomovirus for induction of viral resistance in transgenic plants via gene silencing. *Transgenic Research*.

